# Ocular Changes in Cystic Fibrosis: A Review

**DOI:** 10.3390/ijms25126692

**Published:** 2024-06-18

**Authors:** Slawomir Liberski, Filippo Confalonieri, Szczepan Cofta, Goran Petrovski, Jarosław Kocięcki

**Affiliations:** 1 Department of Ophthalmology, Poznan University of Medical Sciences, A. Szamarzewskiego 84, 61-848 Poznan, Poland; j.kociecki@gmail.com; 2 Department of Ophthalmology, IRCCS Humanitas Research Hospital, 20089 Rozzano, Milan, Italy; filippo.confalonieri01@gmail.com; 3 Department of Biomedical Sciences, Humanitas University, 20090 Pieve Emanuele, Milan, Italy; 4 Center for Eye Research and Innovative Diagnostics, Department of Ophthalmology, Institute for Clinical Medicine, University of Oslo, Kirkeveien 166, 0450 Oslo, Norway; goran.petrovski@medisin.uio.no; 5 Department of Ophthalmology, Oslo University Hospital, Kirkeveien 166, 0450 Oslo, Norway; 6 Department of Respiratory Medicine, Allergology and Pulmonary Oncology, Poznan University of Medical Sciences, A. Szamarzewskiego 84, 61-848 Poznan, Poland; s.cofta@gmail.com

**Keywords:** cystic fibrosis, cystic fibrosis transmembrane conductance regulator, CFTR, eye, mucoviscidosis, ocular changes

## Abstract

Cystic fibrosis (CF), also known as mucoviscidosis, is the most common autosomal recessive genetic disease in the Caucasian population, with an estimated frequency of 1:2000–3000 live births. CF results from the mutation of the cystic fibrosis transmembrane conductance regulator (CFTR) gene localized in the long arm of chromosome 7. The product of CFTR gene expression is CFTR protein, an adenosine triphosphate (ATP)-binding cassette (ABC) transporter that regulates the transport of chloride ions (Cl^−^) across the apical cell membrane. Primary manifestations of CF include chronic lung and pancreas function impairment secondary to the production of thick, sticky mucus resulting from dehydrated secretions. It is well known that CF can cause both anterior and posterior ocular abnormalities. Conjunctival and corneal xerosis and dry eye disease symptoms are the most characteristic manifestations in the anterior segment. In contrast, the most typical anatomical and functional changes relating to the posterior segment of the eye include defects in the retinal nerve fiber layer (RNFL), vascular abnormalities, and visual disturbances, such as reduced contrast sensitivity and abnormal dark adaptation. However, the complete background of ophthalmic manifestations in the course of CF has yet to be discovered. This review summarizes the current knowledge regarding ocular changes in cystic fibrosis.

## 1. Introduction

Cystic fibrosis (CF), commonly known as mucoviscidosis, is the most frequent autosomal recessive genetic disease in the Caucasian population and is caused by mutations in the cystic fibrosis transmembrane conductance regulator (CFTR) gene [1,2]. The estimated frequency of CF in the European population is 1:2000–3000 live births, with the population estimated at 32,000 patients in Europe and 85,000 worldwide [3], with the mean survival age ranging from 44 to 53 years in the developed world [4] and around 20 years in poorer countries [3]. CF was first described as a separate disease in 1938 in the United States by Andersen [5]. The primary observations of CF focused mainly on pancreatic damage. The heatwave that hit New York in 1948 led to the discovery of severe hyponatremic dehydration due to salt loss in sweat and increased understanding of the disease’s pathophysiology as research progressed [6].

Currently, CF is known as a multisystem disease, with the most pronounced manifestation consisting of progressive lung dysfunction with bronchiectasis, pathological remodeling, and fibrosis, as well as chronic gastrointestinal problems with endoexogenous pancreatic insufficiency—disorders affecting these systems are the leading causes of mortality [1]. However, beyond the apparent manifestations, it is known that CF can also affect other organs, including the eye. Primarily, research regarding the ocular changes in the course of CF was focused on vitamin A deficiency in this group of patients and subsequent ocular surface xerosis and abnormal dark adaptation [7]; however, recent research showed that many other structures of the eye may be affected directly or indirectly in the course of the mentioned disease. So far, several ocular changes have been described in patients with cystic fibrosis, affecting both the anterior and posterior segments of the eye.

Due to the expected extension of life length in the CF patient population associated with introducing modulators of the CFTR protein [6], the ophthalmological problems and needs of CF patients require redefinition. Therefore, it is essential to identify and understand ocular changes in this group. This paper summarizes the current knowledge regarding ocular changes in cystic fibrosis.

## 2. Cystic Fibrosis

### 2.1. Molecular Background of Cystic Fibrosis

The CFTR gene was isolated in 1989; this discovery allowed for a much better understanding of the genetic background of CF pathophysiology [6]. So far, approximately 2000 mutations in the CFTR gene, distinguished into six classes (I–VI), have been identified. Mutations that alter the conformation, type of interaction, or stability of the CFTR protein domains determine the onset of CF symptoms and disease severity. The most common mutation of the CFTR gene is the delF508 mutation, belonging to the class II mutation of the CFTR protein, and its prevalence in at least one CFTR gene allele is estimated at 90% among CF patients in the Caucasian population [8].

The CFTR protein is primarily expressed in the apical cell membrane of epithelial cells and is an adenosine triphosphate (ATP)-binding cassette (ABC) transporter that regulates the transport of chloride (Cl^−^) and (HCO_3_^−^) ions secretion across the cell membrane. The active transport of Cl^−^ and HCO_3_^−^ ions from the cytoplasm into the extracellular space is critical for regulating the salt and water balance of epithelial secretions and maintaining their proper hydration levels. Moreover, the insufficient secretion of HCO_3_^−^ ions causes inadequate alkalinization of secretions, which disrupts the function of mucins, enhances the hyperviscosity of secretions, and disrupts the function of bacteriostatic enzymes [8]. The abnormal function of the CFTR protein determines the increased concentration of Cl^−^ ions in body secretions, enabling diagnosis and monitoring of the disease using a sweat test [8,9].

### 2.2. Clinical Findings in Cystic Fibrosis Patients

In the course of CF, abnormalities in the transport of chlorine and bicarbonate ions in the secretory epithelium of the respiratory and gastrointestinal tract, pancreas, vas deferens, and exocrine glands result in the formation of thick, viscous secretions and subsequent recurrent bacterial respiratory infections, leading to persistent respiratory tract dysfunction, pancreatic insufficiency occurring at a young age, gastrointestinal disorders, and infertility in men (Table 1) [1,6,10,11].

Pulmonary impairment is considered the primary clinical manifestation of CF, and respiratory failure is the most common cause of death in CF patients [3]. Ionic imbalance and the hyperviscosity of the respiratory mucus result in decreased mucociliary clearance and promote bacterial colonization and local inflammation. These changes lead to the progressive degradation of the pulmonary epithelium and both lung function and structure.

Nowadays, due to advancements in CF therapy, the average survival time of CF patients has increased [21]. In 2012, the average life expectancy was about 40 years, while in 1938, 70% of children died in early childhood [1]. In contrast, it is predicted that access to modern therapies for CF patients using CFTR protein modulators may enable an extended average survival beyond 70 years [22].

### 2.3. Nutritional Status of CF Patients

It has been established that length and quality of life correlate with CF patients’ nutritional status [23]. Vitamin A and other fat-soluble vitamin deficiencies are associated with fat malabsorption secondary to chronic pancreatic insufficiency in this group of patients. Vitamin A deficiency is believed to be a problem in poor societies in developing countries, while in Western countries, it mainly occurs in predisposed populations, including individuals on fat diets, malnourished patients with systemic diseases, and alcoholics [24]. However, CF patients, due to chronic pancreatin insufficiency and low fat absorption, are also susceptible to vitamin A deficiency and related complications [24]. Some reports reveal that vitamin A deficiency occurs in 10–40% of CF patients [25].

Vitamin A is crucial for the proper differentiation of the epithelial layer and the proper regulation of its secretory functions [26]. In addition, vitamin A is essential for synthesizing rhodopsin in the retina [27]. Hence, the most characteristic symptoms of vitamin A deficiency in CF patients, in addition to intracranial hypertension, are ophthalmic manifestations such as conjunctival xerosis and abnormalities in nocturnal vision [23]. Based on an analysis of the Ebers Papyrus, we can assume that the therapeutic properties of consuming animal liver for night vision disorders and xerophthalmia were already known in ancient Egypt [28]. 

Vitamin A deficiency as the exact cause of night blindness and xerophthalmia in humans was recognized in the course of World War I based on the study of nutritional disorders and symptoms occurring in soldiers who fought in the trenches for long periods [28]. In 1938, Andersen revealed that, based on autopsies of CF children, symptoms of vitamin A deficiency were shown to be present in nearly 20% of this population [5]. It has been shown that CF patients may exhibit reduced serum and tissue vitamin A concentrations despite normal or elevated liver vitamin A concentrations; thus, the conclusion has been drawn that the process of coupling vitamin A to retinol-binding protein (RBP) during mobilization of liver reserves may be abnormal in these patients [26].

Two other nutrients whose levels should be monitored, particularly in CF patients, are vitamin E and zinc. Correct serum vitamin E concentrations are necessary for the proper absorption of vitamin A. Thus, vitamin E concentrations should be monitored, especially in cases of vitamin A deficiency resistant to supplementation [26]. Zinc is a critical micronutrient in releasing vitamin A from RBP and is a coenzyme for retinol dehydrogenase in the retina [27].

## 3. Ocular Surface

### 3.1. Conjunctiva

The conjunctiva is a thin membrane with secretive, absorbent, and protective properties covering the inner part of the eyelids—the palpebral conjunctiva—and the part of the sclera in contact with the external environment—the bulbar conjunctiva [29]. The conjunctiva regulates the transport of electrolytes and, thus, fluid secretion, ensuring the maintenance of an adequate tear film composition that is anatomically and embryologically equivalent to the epithelium of the upper respiratory tract [29]. The presence of CFTR protein in the apical part of the conjunctival epithelium of both the ocular and eyelid conjunctiva in mammals was demonstrated by Turner et al. In the abovementioned study, CFTR protein was found in rabbit, rat, and pig conjunctival preparations using the CFTR-specific antibodies Mab13-1 and G-499, as well as in rabbit bulbar and eyelid conjunctiva using reverse transcription polymerase chain reaction (RT-PCR) [29]. These results confirmed that despite morphological differences between the eyelid and bulbar conjunctiva, chloride ion transport mediated by the CFTR protein acts in corresponding mechanisms [29]. The quantitative contribution of the CFTR protein to chloride ion transport through the conjunctiva has yet to be precisely determined. It is known that, in addition to the CFTR protein, voltage-gated chloride-conducting ion channels (ClC) and chloride channel accessory 2 (CLCA2) are involved in chloride ion transport through the human conjunctiva; in addition, the results of some studies have indicated the presence of a PKA/ATP-regulated outwardly rectifying chloride channel (ORCC) modulated by CFTR [29].

Neugebauer’s study revealed that conjunctival xerosis was found in 10% of CF patients over the age of 12 years, despite chronic supplementation with pancreatic enzymes and vitamin A at 5000 IU daily [26]. In contrast, in a study by Rayner et al., 3 of 52 patients with an average age of 16 years treated with vitamin A at 5000 IU and vitamin E at 100–200 mg daily presented conjunctival xerosis [27]. Petersen described the case of a 16-year-old female patient with abnormal dark adaptation and conjunctival xerosis manifesting as the presence of Bitot’s spots with malabsorption secondary to noncompliance with dietary recommendations and a lack of supplementation of pancreatic enzymes and fat-soluble vitamins. Interestingly, night vision normalized after implementing pancreatic enzyme supplementation, with subsequent resolution of xeropthalmia after the recommended vitamin A augmentation [28].

#### The Role of Pro-Inflammatory Antigens and Mediators

It is well known that inflammatory processes play one of the major roles in the pathogenesis of dry eye syndrome (DES). Recent findings have shown that conjunctival epithelial cells can mediate the inflammatory response by expressing cell adhesion molecules (ICAM-1) that regulate leukocyte migration to the site of the inflammation and class II major histocompatibility complex (MHC) molecules, such as human leukocyte antigen (HLA)-DR, that play a crucial role in antigen presentation and T-lymphocyte activation [10]. The two most commonly used diagnostic tests in DES are the Schirmer test, which evaluates tear production, and the TBUT test, which assesses tear film stability. The Schirmer test is a method of measuring tear secretion using paper strips placed on the temporal side of the conjunctival sac for 5 min. The proximal portion of the strip placed in the conjunctival cul-de-sac moistens by absorbing tears. Three variations of the Schirmer test are known: variation I is performed with the eyes open without prior local anesthesia; variation II involves measuring reflex tear secretion after nasal stimulation; and variation III involves measuring sunlight-stimulated tear secretion. The Tear Film & Ocular Surface Society Dry Eye Workshop II (TFOS-DEWS II) considered less than 10 mm for 5 min as the cutoff value for the Schirmer test in the DES diagnostic criteria [30,31]. Tear film break-up time (TBUT) involves measuring the tear film break-up time following a blink. Improper tear film stability is determined by the inadequate composition of the tear film, which in turn disturbs its viscosity and leads to an abnormal surface tension. TBUT values of less than 10 s are considered abnormal and indicate tear film instability, which is a specific sign of DED [30,31].

Mrugacz’s studies have shown a strong association between pro-inflammatory factors and the occurrence of DES in CF patients [10,32]. Conjunctival impression cytology (CIC) revealed significantly higher HLA-DR expression in CF individuals compared to healthy controls (16.93 ± 10.33% vs. 8.10 ± 1.94%; *p* = 0.0019) [10]. Additionally, there was a substantial reduction in tear film instability index values in the CF group. The mean tear breakup time (TBUT) value in CF patients was 5.3 ± 2.6 s, compared to 9.9 ± 1.2 s (*p* < 0.0001) in controls. Similarly, in the CF patients, the mean Schirmer test result was 9.68 ± 5.54 mm, compared to 25.21 ± 3.08 mm in the control group. Notably, in CF participants, the rate of HLA-DR expression positively correlated with the reduction in the Schirmer test and TBUT values [10].

In another Mrugacz’s study, markedly elevated levels of macrophage inflammatory protein 1-alpha (MIP-1α)—a pro-inflammatory chemokine that mediates the process of tissue infiltration by macrophages—in the tear fluid of CF patients compared to healthy individuals were found [32]. The level of MIP-1α positively correlated with the severity of CF as assessed by the Schwachman scale and correlated negatively with indicators of ocular surface disease—the Schirmer test and TBUT [32]. The other experiment revealed significantly elevated tear fluid levels of interleukin 8 (IL-8) and interferon-gamma (IFN-ỿ), which correlated positively with disease severity and negatively with mild disease severity according to the Schwachman scale. In addition, there was a negative correlation between the values of the Schimer’s test and TBUT results and tear fluid INF-ỿ levels [33].

Results of conjunctival exfoliative cytology (CEC) performed in 40 CF patients showed abnormalities occurring in 20%, 32.5%, 35%, and 12.5% of individuals according to Tseng’s grades I, II, III, and IV, respectively. Interestingly, the severity of digestive disorders measured by the steatocrit index correlated positively with the degree of conjunctival lesions as determined by Tseng’s classification [9]. Sheppard et al. showed no abnormalities, including keratinization of epithelial cells or morphological changes involving the goblet cells; however, the authors highlighted the limited ability to assess goblet cell density due to the insufficient quality of the collected samples [34].

### 3.2. Tear Film and Lacrimal Glands

The tear film is a pre-conjunctival structure about 2–5.5 µm thick made up of three main components, including mucins, water, and lipids, that form three layers: mucous, aqueous, and lipid; however, there is now a trend to classify the mucous and aqueous layers into a single mucous–aqueous layer. The mucin layer comprises glycoproteins—mucins (mainly MUC5-AC) produced by corneal and conjunctival epithelial cells, goblet cells, and Manz’s glands. The primary function of this layer is to create a hydrophilic environment and reduce surface tension. The main functions of the aqueous layer—which consists mainly of water, electrolytes, oxygen, lactoferrin, lysozyme, and VEGF—are to ensure proper hydration of the ocular surface, be a barrier to infection, and supply compounds for accurate corneal metabolism and wound healing. The lacrimal glands and the conjunctival and corneal epithelium principally produce this layer. The lipid layer produced by Meibom’s glands, due to its abundance of cholesterol esters and waxes, protects against evaporation and lowers surface tension [35]. In general, three main functions of the tear film have been highlighted: protecting the ocular surface and providing nutrition to the cornea, as well as contributing to the refractive power of the eye’s optical system [35,36].

CFTR, acting as a Cl^−^ channel, is one of the crucial pro-secretory channels on the ocular surface [37]. Studies in a mouse model of dry eye syndrome showed that CFTRact-K089 (aminophenyl-1,3,5-triazine), a small-molecule activator of human CFTR, restored secreted tear volume to basal levels after topical administration, preventing corneal epithelial dysfunction [38]. Promising results were also obtained in mice with scopolamine-induced dry eye after topical application of Cact-3 (7-(3,4-dimethoxyphenyl)-N-(4-ethoxyphenyl)pyrazolo [1,5-α]pyrimidine-2-carboxamide) and isoharmentin; after treatment, increased tear secretion, reduced expression of pro-inflammatory mediators, and improved ocular surface condition were observed in both studies [38,39].

It was proven that CF patients may present with instability of the tear film [9,40,41]. In Castagna’s study, the values of tear film instability diagnostic tests were abnormal in groups III and IV of Tseng’s scale—significantly lower TBUT values of 11.8 ± 3 and 10.2 ± 2 were observed in groups III and IV of Tseng’s classification compared to 18.4 ± 3 and 15.8 ± 2 in groups I and II and 16.1 ± 2 in the control group [9]. In Morkeberg’s study, a shortened TBUT value was observed in 49% of patients, while a Schirmer test showed less than 5 mm/5 min tear production in 31% of individuals. The Rose-Bengal solution staining showed signs of epithelial damage in 23% of patients. Importantly, all individuals’ serum vitamin A levels were normal [40]. The age differences between both populations may explain the discrepancies in the results of the above studies—in Castagna’s survey, the mean age of participants was 13.5 ± 11 years (range 5–34), while in the study by Morkeberg et al., the study group consisted solely of adult individuals with a mean age of 23 years (range: 18–44 years), as the TBUT time value has been shown to decrease with age [42].

Interestingly, in the Ansaris’s study, in the CF patients with normal vitamin A levels, the indicators of DED—reduced tear film stability and the presence of dying epithelial cells—were found in only 2 of the 28 individuals studied [7], but such a low prevalence of tear film impairment may have been due to the excellent nutritional status of the study group. In Sheppard’s study, there was no significant difference in TBUT values between CF patients and matched healthy controls; however, in this study, there was a deviation from the classic technique of performing the TBUT test by administering local anesthetic drops before the test, which may have significantly affected the results by moistening the ocular surface [34]. The latest study investigating TBUT time in adult CF patients demonstrated a remarkably higher prevalence of shortened TBUT time compared to healthy individuals (44.0 vs. 3.3%) [41]. These findings are consistent with previous studies and confirm that CF patients are characterized by marked tear film instability. However, the exact cause of this condition is not yet known; therefore, further research is needed to elucidate this phenomenon’s background fully.

The lacrimal glands can be involved in cystic fibrosis, which can cause ocular surface abnormalities involving both quantitative and qualitative abnormalities of the tear film. In the report published by Alghadyan, histopathological findings of the lacrimal glands of CF patients were described as reduced amounts of glycoproteins in lacrimal gland secretions and degenerative changes of the acini [43].

#### 3.2.1. Reduced Tear Film Secretion

It has been proven that CF patients may have inadequate tear film secretion. The Schirmer test, used to evaluate tear film secretion in Mrugacz’s study, showed remarkably reduced tear film secretion in 81% of the CF patients, with a mean value of 9.6 ± 5.5 mm compared to 25.0 ± 3.0 mm in healthy individuals [44].

Similarly, in a study by Sheppard and coworkers, CF patients had significantly lower Schirmer test results than matched controls [34]. Another study also showed that the tear secretion in CF patients was significantly lowered at 4.8 ± 3 mm and 4.2 ± 2 mm compared to 22.5 ± 5 mm and 16.3 ± 3 mm in Tseng’s classification groups I and II, as well as 18.7 ± 6 in the control group. In contrast, Kalyaci and coworkers showed no considerable differences between Schirmer test values and the incidence of blepharitis in CF children with a mean age of 6.5 ± 4.2 years in moderate to excellent clinical status according to the Schwachman scale compared to controls. The Schirmer test’s mean value in the study mentioned was 19.1 ± 8.1 mm and 23.1 ± 6.5 mm in the CF and control groups, respectively [45]. Rolando also found no difference between the Schirmer test value in CF patients (31.07 ± 5.86 mm) aged 5–21 years compared to controls (29.07 ± 4.18 mm) [46]. McCannel indicated that the aqueous humor flow rate in CF patients assessed with fluorophotometry was not considerably different from the control values [47]. However, the age difference between the study groups in the cited studies is worth mentioning because tear secretion tends to decrease gradually with age [48]. The low sample size in McCannel’s study may affect the results; therefore, further research is needed to clarify the discrepancies between the available studies.

#### 3.2.2. Changes in Tear Film Osmolarity

In 1958, Di Sant ‘Agnese and coworkers, one of the pioneering groups in CF research, were the first to describe changes in the ionic composition of CF patients’ tear fluid. They found abnormalities concerning the secretion of the exocrine glands involving abnormally high concentrations of electrolytes in sweat, saliva, and tears. Examination of tear composition showed elevated chloride and sodium levels with average potassium ion concentrations compared to healthy individuals [49].

Botelho also evaluated the concentrations of major cations (Na^+^, K^+^, and Ca^2+^) and Cl^−^ anion in the tear fluid and the effect of changing the tear flow rate on their values in CF adolescents. The results revealed that the CF patients and their healthy siblings had similar minimum and maximum tear flow rates. The concentration of Cl^−^ ions was the same in both study groups and did not depend on the flow rate. The concentration of K^+^ ions showed a positive correlation to the flow rate value; however, similar to chloride ions in the assumed flow rate, it did not differ significantly between the CF patients and their healthy siblings. Sodium ion concentrations were markedly different between the study groups. In CF patients, a low flow rate was associated with lower Na^+^ ion concentration compared to controls; however, at the maximal flow rate, Na^+^ ion concentration was markedly higher than in healthy individuals, who did not show flow rate-dependent changes in sodium ion concentration. The levels of Ca^2+^ ions were higher in the CF group, regardless of the degree of tear flow rate [50]. The relationship between tear ion concentrations and flow rate makes it difficult to compare the results of separate studies due to different protocols and tests conducted under nonidentical conditions.

Analysis of tear film osmolarity in 30 CF pediatric patients, with a mean age of 11 years, showed a marked increase compared to the control group. The average tear film osmolarity of CF patients was 315.6 ± 30.5 mOsmol/L, compared to 298.2 ± 16.4 mOsmol/L in healthy individuals. What is worth emphasizing is that tear film osmolarity in CF patients is correlated with the severity of the CFTR gene mutation [51]. Further studies are needed to clarify whether tear osmolality abnormalities are a direct or indirect CF effect.

#### 3.2.3. Role of Abnormal Mucin Expression

Mucins, classified as glycoproteins, compose the mucin layer with a gel-like structure. MUC5AC, essential for maintaining the proper environment of the ocular surface mucin, is the main component of this layer. The mucin layer forms a hydrophilic environment on the ocular surface. Therefore, mucin quantity and quality disorders lead to disruption of ocular surface homeostasis, and a relationship between reduced MUC5-AC and the occurrence of DES has been confirmed [35,52]. Mucin disorders in the tear film of CF patients have not been investigated to date; however, a reduction in the amount of MUC5AC and MUC5B in airway secretions has been confirmed using confocal microscopy by Henke and coworkers [53], which provides a basis for further research to fully clarify this issue.

### 3.3. Cornea

Animal studies have shown that the CFTR gene is expressed in the corneal epithelium and the endothelium [54,55]; however, it is still unclear whether CF may directly affect the corneal tissue. The indirect effect is manifested mainly via CF influence on decreased tear fluid secretion and tear film instability, resulting in chronic problems with maintaining proper ocular surface hydration. The results of the studies completed so far have mainly shown that corneal staining is a common feature of CF. Corneal epithelial defects found after fluorescein staining were present in 82% of CF patients in Sheppard’s study [34]. Botelho and coworkers revealed that corneal staining was present in 60% and 73% of CF children, respectively, compared to 10% and 30% of their healthy siblings [50]. Another study showed a lower occurrence of punctate loss of corneal epithelium in 4 of the 40 (10%) CF patients examined after fluorescein staining [9]. In turn, in a study by Kalyaci in children (mean age of 6.5 ± 4.2), there was no significant difference in the prevalence of corneal epithelial defects compared to healthy individuals [45].

Case reports showed that corneal abnormalities can be the initial sign of CF, especially in infants and early childhood. A case report of a 5-month-old girl with the initial manifestation of CF as xerophthalmia of both eyes leading to bilateral large paracentral opacities and corneal ulcers requiring emergency keratoplasty was described by Wamsley and coworkers [56]. A similar case report of a 2-month-old girl with the initial presentation of CF as bilateral xerophthalmia, keratomalacia, and corneal ulcers caused by Pseudomonas aeruginosa has also been reported. Analogous to the report presented by Wamsley et al., in both cited cases, vitamin A deficiency was found after laboratory testing and was seen as a trigger for the onset of corneal abnormalities [57]. In a 2.5-year-old Iranian boy, bilateral corneal stromal opacities have been noted as one of the first CF symptoms. Further examination revealed elevated chlorine levels in the sweat test and A and D vitamin serum deficits. After multivitamin supplementation, antibiotic therapy, and pancreatic enzyme substitute treatment, corneal opacities gradually reduced during the six-month follow-up [58]. Lindenmuth presented a case of a 16-month-old girl with severe photophobia and observable growth retardation. Ophthalmological examination revealed bilateral corneal xerosis with stromal edema, opacification, and conjunctival and corneal keratinization. Further laboratory tests showed malnutrition along with vitamin A deficiency, and after the determination of sweat chloride levels, the definitive diagnosis of CF was made [59].

Endophthalmitis occurring along with spontaneous corneal perforation with subsequent extrusion of the crystalline lens was described in a 5-month-old girl born prematurely at 33 Hbd. Microbiological examination of conjunctival sac secretions indicated an etiology of Pseudomonas aeruginosa. Further diagnostics revealed the presence of a homozygous delta F508 mutation [60].

Vitamin A deficiency can manifest as rapid corneal necrosis and may appear before conjunctival xerosis and other symptoms of ocular surface damage [24]. Brooks described two cases of 9-month-old infants with corneal abnormalities. In the first case, a Caucasian infant with CF developed peripheral corneal ulceration in the right eye, unresponsive to antibiotic therapy. Laboratory tests showed reduced serum levels of A and E vitamins. A conjunctival biopsy revealed bilateral keratinization and the absence of goblet cells. The corneal ulcers healed after implementing vitamin A supplementation. Electroretinography (ERG) revealed a 50% attenuation of photopic and scotopic responses. The second case referred to a CF male infant with photophobia and eye tearing. Ocular surface examination revealed bilateral diffuse punctate superficial keratitis and conjunctival xerosis. A conjunctival biopsy showed keratinization and the absence of goblet cells. Based on a general clinical picture, including malnutrition and failure to thrive, as well as an ophthalmologic examination, a diagnosis of CF was suspected and was further confirmed by laboratory tests. After implementing vitamin A supplementation, the ocular surface condition returned to normal within a few days. Similar to the first case, abnormal scotopic and photopic responses were noted without improvement during examination [61].

On the other hand, Morkeberg showed that dry eye-specific signs, including snake-like chromatin and parakeratotic cells, were present in 32% of patients despite normal serum vitamin A levels, implying that ocular surface abnormalities may also be a primary manifestation of the disease [40].

## 4. Lens

The lens, from its development at around 28 days’ gestation and the formation of the lens placode, undergoes continuous remodeling throughout life, involving the movement of old cells to the center of the organ, which allows maintaining metabolically active cells in the cortical layer. Such continuous rebuilding provides the appropriate refractive properties necessary to focus the image on the retina and minimize spherical aberration. On the other hand, it makes the lens susceptible to undesirable effects—such as presbyopia as well as cataracts, which can be caused by aging processes and the influence of cataractogenic factors [62].CF patients may present with reduced lens transparency. This condition may result from metabolic abnormalities in the course of CF. However, current research into new therapies for CF has also indicated an association between the use of CFTR protein modulators and an increased occurrence of lens opacification in the pediatric population [63].

### 4.1. Reduced Lens Transparency

An association between markedly reduced lens transparency, measured with the OLM 701 device, and the incidence of CF in patients aged 5–34 years (mean age: 13.5 years) has been demonstrated. Noteworthy, individuals with severe nutritional deficiencies with a high steatocrit index had substantially reduced lens transparency compared to patients with better gastrointestinal function and controls [9,11].

Several potential mechanisms that may explain CF patients’ susceptibility to more rapidly occurring lens opacities have been proposed. The main hypothesis is that due to vitamin A and E deficiency, there is a secondary reduction in the concentration of vitamin C in the aqueous fluid in the lens, resulting in diminished antioxidant activity and an accelerated opacification of the lens. This hypothesis is supported by the evidenced contribution of vitamin A to the regulation of lysosomal membrane permeability, which affects the release of hydrolytic enzymes into the ciliary epithelium and determines the transport of vitamin C into the aqueous. The second mechanism involves decreased mucopolysaccharide metabolism and stabilization of the lysosome-8-containing acid hydrolytic enzyme, which leads to swelling of the ciliary body and acceleration of lens opacification [9,11].

### 4.2. Effect of CFTR Protein Modulators on Lens Transparency

A preclinical animal study has shown an incidence of cataracts in immature rodents exposed to—the CFTR modulator, ivacaftor. In juvenile rats, lens opacification was observed after exposure to ivacaftor on postnatal days 7–35 with dose levels of 10 mg/kg/day and higher (an equivalent of 0.1–0.8 of the maximum recommended human dose). Interestingly, no cases of lens opacity were observed in rat fetuses whose mothers received ivacaftor from days 7–17 of gestation, as well as in rats fed up to 20 days after birth by mothers treated with ivacaftor. In addition, these findings were also not present in mature rats or 3.5–5 month-old dogs exposed to ivacaftor [64,65].

Similarly, cases of lens opacification in the pediatric population following ivacaftor, both as monotherapy and in combination, have also been reported. Data from published studies have shown that the incidence of cataracts in the pediatric population ranges from 0.57% to 4.17% [63].

Current research has shown that in CF patients aged 2–6 years treated with ivacaftor, cortical cataracts occurred in 4.17% of participants within 84 weeks of treatment initiation, while in the age group over 12 years, the presence of posterior subcapsular cataracts was observed in 0.57% of individuals within 96 weeks of initiating ivacaftor therapy [63,66]. Another study reported the presence of cataracts of unspecified type in 1.72% of CF patients aged 6–11 years within 24 weeks of starting treatment with the ivacaftor–lumacaftor combination, as well as in 1.54% of individuals over 6 years after treatment with the ivacaftor–tezacaftor combination [63]. However, it is noteworthy that in some of the reported cases, exposure to other factors that may promote lens opacification (e.g., the use of systemic corticosteroids and exposure to radiation) was also present [64]. There are also concerns regarding the use of ivacaftor during pregnancy and the potential risk of congenital cataracts in newborns. Jain et al. reported three cases of bilateral congenital cataracts in a case series consisting of 23 pregnant and lactating women treated with the elexacaftor–tezacaftor–ivacaftor combination. It is worth emphasizing that no other cataract-promoting underlying conditions were found in this case [67].

To date, the exact mechanism underlying the occurrence of lens opacification in both rats and humans has yet to be fully understood [63], and this issue requires further research. Initial and follow-up ophthalmic examinations of CF patients, especially in the pediatric population, as well as pregnant and breastfeeding women receiving CFTR modulators, are crucial to further determining the potential association between the use of these drugs and the incidence of lens opacification.

## 5. Posterior Segment of the Eye

### 5.1. Retina

Within the human retina, CFTR gene expression has been evidenced in the retinal pigment epithelium (RPE) layer [68]. RPE is a critical part of the retina and consists of a single layer of pigmented cells located between the neural retina and the choroid, being part of the blood–retinal barrier (BRB). The RPE has multifaceted functions, including glucose and ion transport from the choroid to the photoreceptors, phagocytosis of unnecessary metabolic products from the photoreceptor’s outer segments, and preventing the accumulation of waste metabolic products. Moreover, the RPE plays a vital role in the retinol cycle [69]. In addition, melanin accumulated in RPE cells can absorb excess light, serving a protective function for photoreceptors [69,70]. These attributes make the proper structure and function of the RPE layer essential for preserving the normal process of vision.

An electroretinogram (ERG) is a well-known method to assess the electrophysiological function of the retina and a noninvasive test used to diagnose many conditions [69]. Assessment of RPE function in the ERG test is performed by evaluating the c-wave, oscillatory potentials (OPs), and light peak (LP) [71]. The c-wave results from decreased subretinal potassium ion (K^−^) concentration, causing hyperpolarization of RPE cells and receptors [72]. OPs are negative deflections of the curie following the c-wave and result from hyperpolarization of the RPE basement membrane caused by an influx of chloride ions. At the same time, the light peak (LP) is the result of the RPE basement membrane depolarization secondary to an increase in chloride ion (Cl^−^) conductance [73].

In an animal study by Wu and coworkers, abnormalities in ERG recordings were found in CFTR-mutant mice with the Fdel508 mutation (CFTR^+/−^) consisting of reduced amplitudes of c-wave, LP, and FO—RPE-dependent components of ERG recordings compared to healthy individuals (CFTR^+/+^), which may suggest a link between the Fdel508 mutation and abnormal RPE function. However, the results did not meet the statistically significant level, probably due to the small sample size of mice with homozygous mutations. Importantly, unlike the RPE-dependent components of the ERG recording, the a-wave was normal in both the Fdel508 group and the control group [71], demonstrating the lack of effect of the del508 mutation in the CFTR gene on photoreceptor function due to the a-wave reflecting photoreceptor function in the outer retina [74]. Furthermore, an analysis performed on a small group of mice lacking the CFTR gene in both alleles (CFTR^−/−^) showed similar changes in the c-wave, FO, and LP to those found in mice with Fdel508 (CFTR^+/−^), suggesting that CFTR is involved in the generation of these three components of the ERG; however, they are not entirely dependent on it either; it may not be excluded that other chloride channels ClC and CLCA are also involved in the generation of these components [71].

In the case report by Suttle and Harding, abnormal results of the ERG and VEP tests were recorded in Caucasian infants prior to the confirmed CF diagnosis. The baseline ERG recording analysis showed a normal photopic and fast-flicker response with no scotopic response. Similarly, there were no VEP responses. After the CF diagnosis and supplementation with vitamins A, D, E, K, B1, B2, and C were initiated, the scotopic recording returned to normal; however, the VEP recording remained abnormal. These findings suggest the importance of normal levels of vitamin A in the body for proper dark vision adaptation and prove the possibility of improving the scotopic response after implementing supplementation and compensating for nutritional deficiencies. Persistently abnormal VEP recordings with normal photopic and scotopic responses may indicate a functional disorder of the visual pathway located distal to the photoreceptors [75].

Severely reduced plasma concentrations of two carotenoids—lutein and zeaxanthin—correlating with reduced macular pigment optical density (MPOD) assessed using heterochromatic flicker photometry were found in adult CF patients compared to healthy individuals. Interestingly, visual functions such as color discrimination and retinal function (assessed by mfERG), as well as contrast sensitivity, showed no significant differences compared to the control group. Plasma tocopherol concentrations were normal in the CF patients; however, lycopene and B-carotene levels were reduced despite supplementation in all study participants. Plasma lutein and zeaxanthin were about 47% and 36 of the concentrations in healthy individuals, respectively. Interestingly, both groups showed a significant correlation between plasma concentrations of lutein and zeaxanthin and MPOD. Despite low plasma levels of antioxidants, macular status in all CF patients was normal, and retinal abnormalities secondary to the central retinal vein occlusion (CRVO) were seen in one patient [76]. Additionally, one case of a full-thickness macular hole and one case of a macular cyst among 24 CF patients were described in an observational study by Bruce et al. [77]. In Hiscox’s study, there was also a case of a 69-year-old male CF patient with dry age-related macular degeneration (AMD) signs presented as drusens and another case of the presence of epiretinal membrane (ERM) in a 31-year-old female CF patient [78]. Morphological analysis of the retina in CF patients using optical coherence tomography (OCT) scans showed a reduction of retinal thickness in the macula, especially the photoreceptor/RPE layer, compared to healthy individuals. Hiscox concluded that it could be a sign of premature aging of these structures, developing AMD, or a disruption in the formation of these layers secondary to fat absorption disturbances. Interestingly, these findings were not observed in CF-related diabetes [78]. Therefore, the role of systemic factors in CF patients associated with retinal pathologies should be under the special attention of researchers because of CF patients prolonged life expectancy and the possible increased risk of developing retinal pathologies.

### 5.2. Optic and Oculomotor Nerve

Nerve tissue, due to its high rate of oxygen consumption, is susceptible to insufficient oxygen supply. Chronic hypoxemia resulting from lung disease may lead to multiple degenerative changes within the nervous system [1]. Retinal tissue is known to be more sensitive to hypoxia than brain tissue due to its higher energy dependence [79]. Impaired vision or even complete loss of vision secondary to severe systemic hypoxia has been reported. Hypoxic damage to RGCs and their axons that form the optic nerve is critical for transmitting visual information to brain structures that are part of the visual pathway. Despite these structures, hypoxia can also damage glial cells, especially oligodendrocytes [79].

Giannakouras and coworkers showed that CF patients may present anatomical signs of early optic nerve damage. In their study, reduced inferior-nasal retinal nerve fiber layer (RNFL) thickness in CF was observed; moreover, CF patients with the homozygotic F508del mutation had lower RNFL thickness in the interior-nasal quadrant compared to other CF patients [41].

A pilot study with a cohort of 11 CF patients proved a correlation between optic nerve functional parameters and pulmonary function tests [1]. In the cited study, an unconventional perimetry test of the Frequency-Doubling Technology (FDT) was used to assess the effects of recurrent episodes of hypoxia and hypercapnia on optic nerve function and retinal GC and RNF layers in CF patients aged 30 ± 13 years and with moderate pulmonary dysfunction with a mean FEV1 of 58%. The FDT perimeter aims to screen for visual field abnormalities. The mean deviation (MD), which indicates overall field depression, and the pattern standard deviations (PSD) intended for assessing focal field depression are the primary indexes for the visual field analysis provided by the FDT perimeter. The results showed a marked correlation between the MD of FDT and hematocrit (Ht) and FEV1 and FVC values; in addition, a significant correlation was also noted between PSD, Ht, SpO_2_, FEV1, and FVC, which may suggest an effect of chronic hypoxia on insufficient blood supply and oxygen supply to the inner retinal layers, leading to damage to the GC and RNF layers [1].

Spaide examined 32 CF patients aged 19.9 ± 9.2 years to assess optic nerve pathway function on VEP testing, contrast sensitivity testing, color vision, and pupil reactivity assessment after mydriatic administration [80]. Six of the 17 patients using prior chloramphenicol had binocular (5 participants) or monocular (1 participant) VEP latencies greater than 2SD; moreover, all patients with binocular VEP abnormalities presented color vision disturbances when tested with Ishihara plates and a worse contrast sensitivity test. The mean P100 wave latency was significantly higher in patients with a history of chloramphenicol use than in patients not treated with this drug (107.6 s vs. 96.9 s) [80].

Examination of pupillary reactivity showed abnormalities in pupillary reactivity in the form of partial or complete oculosympathetic paralysis were detected in 8 of 23 patients; significantly, the presence of impaired pupillary reactivity correlated with the disease severity assessed with the use of the Schwachman scale [80].

### 5.3. Reduced Contrast Sensitivity and Abnormal Dark Adaptation

It is believed that photoreceptor function, due to its dependence on the retinol cycle, is disrupted at the earliest in cases of vitamin A deficiency in the body, which can manifest as reduced contrast sensitivity or abnormal dark adaptation [81].

#### 5.3.1. Reduced Contrast Sensitivity

Vitamin A deficiency may produce abnormal contrast sensitivity. A case report of a 16-year-old male CF patient with loss of contrast sensitivity has been reported by Leguire and coworkers [81]. During the presentation, visual acuity was 20/30 in both eyes, and conjunctival xerosis manifested as the presence of Bitot’s spots. ERG results showed reduced b-wave amplitude bilaterally. Laboratory tests revealed serum retinol below 10 µg/dL with a reference range of 30–95 µg/dL. After four months of vitamin A supplementation with a dose of 25,000 IU/day, serum vitamin A levels returned to a normal range, along with ERG and contrast sensitivity test results [81].

However, despite the important role of vitamin A in contrast vision, both CF patients with normal and abnormal serum vitamin A levels can present abnormalities in contrast sensitivity. Morkeberg showed that 19 of 35 adult CF patients presented reduced contrast sensitivity despite normal serum vitamin A levels in all individuals [40]. Similarly, in a study by Ansari et al., contrast sensitivity was abnormal in 8/28 patients with normal serum vitamin A levels [7]. In Spaide’s study, contrast sensitivity was significantly lower in CF patients compared to controls; importantly, CF individuals with a positive history of chloramphenicol use had significantly lower contrast sensitivity at 11.4 and 22.8 cycles per degree compared to those who did not use chloramphenicol [80].

It has been shown that, in addition to nutritional status, a history of chloramphenicol use may also be associated with abnormal contrast sensitivity in CF patients. Chloramphenicol is an older generation antibiotic widely used in the previous decades, and it is now being used with growing frequency due to increasing bacterial resistance to new generations of antibiotics. In addition to its adverse effects on the bone marrow, chloramphenicol has shown time- and dose-dependent negative effects on the optic nerve, potentially leading to toxic optic nerve neuropathy (TON). Chloramphenicol is believed to inhibit mitochondrial protein synthesis, leading to mitochondrial respiratory chain dysfunction and disrupting B vitamin metabolism [82]. Animal studies using a mouse model have shown that a short period of high-dose chloramphenicol therapy can lead to optic nerve damage at the ultrastructural level [83]. These alternations may lead to bilateral visual impairment with central or cecocentral scotoma and color vision abnormalities [84].

#### 5.3.2. Abnormal Dark Adaptation

Vitamin A is essential for rhodopsin synthesis within the retina, and one of the characteristic ophthalmic manifestations of systemic vitamin A deficiency is night blindness, also known as nyctalopia [27]. Several studies have shown that CF patients can present with abnormal night vision.

Neugebauer showed that 6 of 31 CF patients aged over 12 years had abnormal dark adaptation despite supplementation with pancreatic enzymes and vitamin A at a dose of 5000 IU per day. The analysis showed that these symptoms were more common in individuals with serum vitamin A concentrations below 30 µg/dL compared to patients with higher vitamin A levels and healthy individuals serving as controls. Interestingly, there was no relationship between abnormal dark adaptation, the severity of CF as assessed by the Schwachman scale, and the presence of liver dysfunction [26]. A study by Rayner et al. revealed abnormal dark adaptation in 8/52 patients with a mean age of 16 years receiving pancreatic enzyme supplementation, as well as augmentation of vitamin A and vitamin E. In this group, six patients had reduced vitamin A and RBP levels compared to controls [27]. In the group of patients with ocular symptoms, liver dysfunction was significantly more frequent than in asymptomatic patients; also, serum vitamin A and RBP were significantly reduced compared to the asymptomatic CF patients and healthy individuals. There was no association between disease severity assessed by the Schwachman scale, lung function, the Chrispin-Norman score, and either expected height and weight or the frequency of ophthalmic symptoms [27]. In four patients, dark adaptation tests were repeated after a single dose of 100,000–200,000 IU of vitamin A, and in three patients, the results returned to normal, while one patient required further, longer treatment with vitamin A before symptoms resolved [27].

Vernon presented a case series of three CF patients with asymptomatic conjunctivitis, along with co-occurring abnormal dark adaptation in two of them. Common laboratory abnormalities in these patients were serum vitamin A and RBP deficiencies; one individual had abnormal liver tests [24]. Another study evaluated biochemical parameters, including plasma levels of retinol, RBP, zinc, and alpha and beta carotene, and their association with dark adaptation in CF patients. The experiment was conducted on adolescents (mean age of 14.3 years) in good to excellent clinical condition. The results showed that serum levels of retinol and RBP were below the normal range in 9 out of 10 patients; similarly, α and β-carotene concentrations were also reduced. Zinc and vitamin E levels were normal. Although there was no clear correlation between serum retinol levels and dark adaptation test results, the most remarkable dark adaptation abnormalities were observed in the two patients with the lowest serum retinol levels [23]. A study by Ansari et al. showed that adolescent and adult CF patients with normal serum vitamin A levels can present a normal dark adaptation. In this study, CF patients had excellent nutritional status and were provided with an augmentation of 12,000 U/day of vitamin A and 7500 U/kg/day of lipase. All patients had normal serum vitamin A levels, while borderline zinc and retinol-binding protein (RBP) levels were found in four patients. Dark adaptation was normal in all examined individuals compared to the control group and did not correlate with plasma retinol, zinc, and RBP concentrations [7]. Hiscox revealed abnormalities in dark adaptation in patients with cystic fibrosis-related diabetes (CFRD) with a tendency to alleviate after oxygen administration. Interestingly, there were no abnormalities in the overall CF group compared to healthy individuals. These findings should concentrate further research, in particular, on assessing retinal status in CFRD patients to clarify whether they are more susceptible to dark adaptation abnormalities than the general CF population.

## 6. Changes of the Retinal and Choroidal Vasculature

The negative effects of systemic changes on the cardiovascular system in patients with CF have been well documented [85,86]. MacNee demonstrated increased central arterial stiffening, an indicator of premature vascular aging observed in children with CF by Buehler [87]. Moreover, animal studies have shown that mice lacking in both alleles of the CFTR gene are characterized by systemic vascular stiffness as well as higher day and night SBP, DBP, and MDP values compared to healthy mice [88].

Another study showed that overexpression of the CFTR gene in ApoE^−/−^ mice via inhibition of the nuclear factor-kappa B (NF-κβ) pathway and activation of MAPKs leads to reduced vascular inflammation and atherosclerosis progression and inhibits the infiltration of T lymphocytes and neutrophils into atherosclerotic plaques, promoting their stability [89]. An experiment in an animal and human model of pulmonary hypertension (PA) has revealed that CFTR gene expression is significantly lower in vascular endothelial and pulmonary artery smooth muscle cells. Moreover, long-term inhibition of CFTR expression in rats stimulates pulmonary vessel neomuscularization and reduces their relaxation [90]. Other studies have shown that vascular endothelial growth factor (VEGF), a critical factor in both physiological and pathological vessel growth and vascular remodeling, is elevated in the serum of CF patients [2,21]. According to current studies, elevated serum VEGF levels correlate with the severity of retinal vasculopathy [91]. There is no current research regarding ocular vascular changes in CF patients. In 1960, Bruce et al. presented the results of examining 24 CF patients and revealed that morphological vascular abnormalities were present in all 23 patients in whom a fundus examination was carried out. The most common vascular changes were venous engorgement, tortuosity, and dilatation—at least one of them was seen in 20/23 patients, while retinal hemorrhages were found in 9 patients [77]. The case report of branch retinal vein occlusion (BRVO) with secondary macular edema in a 35-year-old male CF patient with elevated serum fibrinogen levels was described by Starr et al. [92]. Hiscox et al. described a similar case of a 35-year-old male CF patient with BRVO and hyperfibrinogenemia [93]. A case of CRVO recurrence in the contralateral eye four years after the first episode in a 31-year-old CF patient with a hypercoagulopathy secondary to increased serum fibrinogen and gammaglobulin levels has also been reported [94]. Another case report concerned the disclosure of pathologic macular lesions, including drusen deposits and subretinal hemorrhages, during fundoscopy in an adult CF patient. Macular OCT and OCT-based angiography showed macular neovascularization (MNV) in both eyes with coexisting subretinal fluid in the right eye [95]. Although little is known regarding the vascular status of the retina and uvea of CF patients, findings of systemic vascular changes indicate that this group of patients should undergo regular fundus examinations, as well as OCT and OCT angiography, to be able to detect possible vascular complications. The retinal and choroidal vascular status of CF patients should be further investigated to fully clarify this issue.

## 7. Management of CF-Related Ocular Complications

Although CF newborn screening (NBS) to detect CF in newborns is a widespread screening program worldwide, this test is still not performed routinely in some countries [96]. As described in Section 3.3, there have been case reports of severe corneal disorders, including severe xerophthalmia, corneal opacities, keratomalacia, and corneal ulcers, manifesting as the first symptom of CF. Therefore, a differential diagnosis should include CF in cases of corneal disorders in children without prior CF NBS. Nonsevere corneal abnormalities, such as keratinization, can be reversible after supplementation, especially with vitamin A and nutritional compensation [97]. Corneal ulcers require the implementation of intensive topical treatment with fluoroquinolones and often systemic empiric antibiotic therapy, while, after revealing the pathogenic factor in culture, targeted antibiotic therapy should be implemented; however, in severe cases—not amenable to pharmacological treatment—corneal keratoplasty is necessary [98].

Dry eye syndrome requires regular ophthalmologic care to minimize adverse effects on the ocular surface and reduce adverse effects on quality of life. The management of patients with dry eye syndrome is discussed in detail in the TFOS-DEWS II Guidelines and, according to the recommended staging of interventions, may include patient education, topical lubricants, tear point occluders, topical corticosteroids of shorter or longer duration, or surgical procedures (e.g., amniotic membrane grafts, surgical punctal occlusion, or rarely tarsoraphy and salivary gland transplantation) [31]. The results of the studies discussed in Section 3.2 of this article showed that DES could result in CF patients with a background of nutritional insufficiencies, especially vitamin A deficiency; thus, in treatment-resistant cases, the patient’s nutritional status should be thoroughly assessed.

The cases of lens opacification reported to date in CF patients can occur both in the course of the disease, exposure to cataractogenic agents (radiation, exposure to systemic corticosteroid treatment), and related to the use of CFTR modulators. In the majority of reported cases, lens opacification did not cause clinically significant visual impairment [35,36,60,61,62,63,64]; however, given the favorable impact on life expectancy of CF patients undergoing treatment with CFTR modulators, the development of cataracts leading to a significant decrease in visual acuity in some patients seems to be inevitable—in these cases, phacoemulsification of cataracts along with implantation of an intraocular artificial lens as the method of first choice for cataract treatment in the general population will be required [99].

Similarly, given the projected increase in life expectancy of CF patients, they should undergo regular fundus examinations for early detection and the ability to implement appropriate management for identified retinal, as well as retinal and/or uveal vessel-related complications. The management of retinal complications in CF appears to be identical to that of the general population and depends on the condition diagnosed.

In the case of abnormal dark adaptation and abnormal sense of contrast reported by CF patients, the nutritional status of the patient, especially vitamin A, should be verified since appropriate supplementation may lead to the reversal of symptoms in the presence of an identified nutritional deficiency. It is worth mentioning that abnormal contrast sensitivity in CF individuals with a positive history of chloramphenicol use may be the result of toxic optic neuropathy, so the use of this drug should be recorded in the patient’s medical history [84].

Due to the possible impact of CF on both anterior and posterior ocular structures, CF patients should undergo regular, complete ophthalmologic examinations to make a diagnosis and implement appropriate management. Due to CF specificity, nutritional status should be evaluated in diagnostically difficult cases or when standard treatment is ineffective.

## 8. Conclusions

Ocular changes in CF can occur in both the anterior and posterior segments of the eye. They can be both a direct and indirect manifestation of the disease. Ocular surface abnormalities in infants may be an initial CF manifestation, especially in countries without routine CF newborn screening. In treatment-resistant cases involving both xerophthalmia, reduced contrast sensitivity, and abnormal dark adaptation, nutritional status should be thoroughly evaluated, especially vitamin A and other fat-soluble vitamin deficiencies. Lens opacification in rodents and children treated with CFTR modulators, as well as cases of congenital cataracts in children of mothers treated with CFTR modulators during pregnancy, have been described; therefore, this issue should be under special attention by clinicians and researchers. Little is known regarding retinal changes and retinal and choroidal vessels in CF, but given the documented systemic vascular changes in this disease, CF patients should undergo regular fundus examinations. A better understanding of ocular changes in the course of CF may improve the monitoring and management of CF patients. As the life expectancy of CF patients increases with advances in therapy, ophthalmic care for CF patients will be a challenge for clinicians in the coming decades.

## Figures and Tables

**Table 1:** Summary of the main systemic manifestations of cystic fibrosis (CF).

Organ System	Affected Organ	Manifestation	References
Respiratory system	Lungs	Chronic infections (bacterial and fungal) and inflammation secondary to the accumulation of the thick, sticky mucus Hemoptysis Bronchiectasis Respiratory failure	[12]
	Paranasal sinuses	Chronic sinusitis Nasal polyps	[13]
Digestive system	Pancreas	Reduced secretion of pancreatic enzymes via duct obstruction resulting in nutrient malabsorption of micro- and macronutrients Pancreatitis Chronic pancreatic inflammation CF-related diabetes	[14]
	Liver	Neonatal jaundice CF-related liver disease (increased viscosity of biliary secretion and secondary cholestasis) Fatty liver Primary sclerosing cholangitis (PSC) Cirrhosis	[14,15]
	Gut	Constipation Diarrhea Meconium ileus	[14,16]
Reproductive system	Genitals (female)	Reduced fertility	[17]
	Genitals (male)	Congenital bilateral absence of vas deferens Azoospermia Infertility	[18]
Skin and skin appendages	Sweat glands	Salty sweat Dehydration	[19]
Skeletal	Bones	Osteopenia Osteoporosis	[20]
	Joints	CF-related arthropathy	[20]
